# A Wearable Electrochemical Sensor Based on a Molecularly Imprinted Polymer Integrated with a Copper Benzene-1,3,5-Tricarboxylate Metal-Organic Framework for the On-Body Monitoring of Cortisol in Sweat

**DOI:** 10.3390/polym16162289

**Published:** 2024-08-13

**Authors:** Pingping Tang, Feiyu He

**Affiliations:** 1 School of Materials and Chemistry, Southwest University of Science and Technology, Mianyang 621010, China; sara@ustc.edu.cn; 2 Engineering Research Center of Biomass Materials, Ministry of Education, Mianyang 621010, China

**Keywords:** molecularly imprinted polymer, metal–organic framework, sweat cortisol, electrochemical sensor, wearable sensor

## Abstract

Owing to their potential to transform traditional medical diagnostics and health monitoring, wearable biosensors have become an alternative evolutionary technology in the field of medical care. However, it is still necessary to overcome some key technique challenges, such as the selectivity, sensitivity, and stability of biometric identification. Herein, a novel, wearable electrochemical sensor based on a molecularly imprinted polymer (MIP) integrated with a copper benzene-1,3,5-tricarboxylate metal–organic framework (MOF) was designed for the detection of stress through the on-body monitoring of cortisol in sweat. The MOF was used as the substrate for MIP deposition to enhance the stability and sensitivity of the sensor. The sensor consisted of two layers, with a microfluidic layer as the top layer for spontaneous sweating and a modified electrode as the bottom layer for sensing. The sensor measured cortisol levels by detecting the current change that occurred when the target molecules bound to the imprinted cavities, using Prussian blue nanoparticles embedded in the MIP framework as the REDOX probe. The proposed sensor exhibited a linear detection range of 0.01–1000 nM with a detection limit of 0.0027 nM, and favorable specificity over other analogies. This facile anti-body free sensor showed excellent stability, and can be successfully applied for in situ cortisol monitoring.

## 1. Introduction

Stress is widely believed to cause various mental health illnesses and life-threatening medical conditions [1,2,3]. Chronic exposure to stress can lead to cardiovascular disease, anxiety, and depressive episodes, as well as risky behaviors [4,5,6]. As stress levels increase, researchers have focused on developing effective sensing technologies which operate through the sensitive and selective detection of related biomarkers to monitor stress [7,8]. Some substances in biological fluids, such as sweat, saliva, and tears, are biomarkers showing human stress levels, including cortisol, dopamine, and histamine [9,10,11]. Among them, cortisol is of great importance for regulating emotional stress, and an increase in cortisol levels is associated with psychological pressure [12,13]. Cortisol concentration in the body fluctuates in a circadian rhythm, being highest early in the morning (5–30 nM), moderate during the day, and lowest at night (<2 nM). The average level of cortisol in sweat is between 0.02 and 0.5 μM. Therefore, the selective, sensitive, and real-rime monitoring of cortisol in biological fluids has clinical implications in predicting the occurrence of stress disorders and managing stress-related diseases [14]. Several technologies have been developed for sweat cortisol determination, such as electrochemical sensors [15,16], the enzyme-linked immunosorbent assay (ELISA) [17], and high-performance liquid chromatography [18]. Among them, electrochemical sensors are excellent for rapid analysis and simple operation while maintaining sufficient sensitivity and specificity. Furthermore, they also possess the abilities to carry out real-time detection and continuous monitoring of pressure levels [19,20]. However, current electrochemical sensors, mostly with biomolecules and aptamers as the sensing unit, tend to be environmentally unstable and thus have a short service time [21,22]. 

A molecular imprinting polymer (MIP) is a kind of functional biomimetic molecular receptor, which has predetermined selectivity and excellent stability as an artificial antibody [23,24,25]. It is a synthetic material that is known to be a highly selective receptor for a range of analytes. An MIP can be easily synthesized by chemical or electrochemical procedures. In these polymerization processes, different quantitative and qualitative variables are closely related, thus influencing the MIP’s effective recognition ability and sensitivity to targets during rebinding. In recent years, various strategies for optimizing MIP synthesis have been explored, including structured experimental design (DoE) [26] and one factor at a time (OFAT) [27,28]. Among them, the DoE is a formal structured approach designed to solve complicated problems where multiple variables interact to realize responsive solutions.

Furthermore, the combination of nanomaterials with the MIP can provide a larger surface area and more accessible imprinting sites to effectively identify target molecules, resulting in a significant improvement in recognition efficiency [29,30]. In recent years, MIPs have been combined with metal–organic frameworks (MOFs) to synergize the advantages and efficiencies of these two materials [31,32,33]. MOFs are three-dimensional network frameworks made up of hybrid polymer materials with adjustable mesoporous structures and variable morphologies, resulting in regular porosity with a high surface area and low density. Due to these excellent properties, they are widely used for sensors [34], separation [35], catalysis [36], and other fields [37,38]. On the one hand, MIPs can greatly improve selectivity by forming imprinting sites with specific molecular functional groups and stereo-chemical structures [39]. On the other hand, MOFs can greatly increase the specific surface area and the number of recognition sites, due to porous octagonal crystal structures [40]. Therefore, the hybridization of MIP and MOFs can uniquely enhance the strength and produce excellent materials with high selectivity, sensitivity, and porosity. 

Inspired by these factors, a novel wearable electrochemical sensor based on the combination of an MIP with MOF is reported, which is designed to realize the in situ and real-time monitoring of sweat cortisol levels. For the preparation the sensing electrode, after the direct synthesis of copper benzene-1,3,5-tricarboxylate (Cu-BTC) on a glassy carbon electrode (GCE) surface, an MIP film was produced by in situ electro-polymerization using o-phenylenediamine (o-PD) as the functional monomer and cortisol as the imprinting molecule under optimized conditions. The MIP produced abundant amino groups that can selectively bind to cortisol molecules through hydrogen bonding. In addition, during the electro-polymerization process, Prussian blue nanoparticles were modified in the MIP film as “built-in” REDOX probes to perform in situ detection. Finally, the modified sensing electrode was fixed on a flexible microfluid chip to create the wearable sensor. This novel sensor can automatically collect sweat from human skin and transmit it to the MOF/MIP-based sensing electrode. This method is capable of the real-time monitoring of sweat cortisol with a low detection limit and exhibited an excellent performance in terms of selectivity and reliability.

## 2. Materials and Methods

### 2.1. Materials

Cortisol, o-phenylenediamine (o-PD), ethylene glycol dimethacrylate (EGDMA), Prussian blue nanoparticles, and 1,3,5-benzenetricarboxylic acid (H_3_BTC) were obtained from Sigma-Aldrich (Shanghai, China). Copper sulfate salts, (NH_4_)_2_S_2_O_8_, potassium ferricyanide (K_3_[Fe(CN)_6_], 99%), potassium ferrocyanide (K_4_[Fe(CN)_6_], 99%), and potassium chloride (KCl) were purchased from Sinopharm Chemical Reagent Co., Ltd (Shanghai, China). Estrone, dehydroepiandrosterone (DHEA), progesterone, estradiol, glucose, estriol, serotonin, dopamine, ascorbic acid, and the ELISA kit for cortisol were all purchased from Aladdin (Shanghai, China). The artificial sweat (acidic, ATTS standard) was obtained from Shanghai Bestbio Biotechnology Co., Ltd (Shanghai, China). Unless otherwise specified, all chemicals and solvents were of reagent grade and used without further purification.

### 2.2. Instruments

The density functional theory (DFT) calculations were handled by the Gaussian 09 program package (Gaussian 09, Revision d.01). A ZETA-20, Z10002-1–1 optical profilometer (KLA Instruments, Milpitas, CA, USA) was used to characterize the contours and coarseness of the electrode surfaces. Cyclic voltammetry (CV) and differential pulse voltammetry (DPV) were performed by an electrochemical analyzer model CHI608E (CH Instruments, Inc., Austin, TX, USA), which was equipped with a typical three-electrode system composed of prepared GCE/MOF/MIP as the working electrode, Ag/AgCl-saturated KCl electrode as the reference electrode, and Pt as the auxiliary electrode. A computer with PsTrace software (5.9) for Windows was used for instrument control and data acquisition. 

### 2.3. Computational Studies and Energy Calculations

Under the framework of DFT, the optimal hybrid functional ωB97XD- was used to optimize the structure of the suggested compound at a set base level of 6–31 G (d,p). The theoretical level of ωB97XD/6–31 G (d,p) was ideal for geometric optimization and the calculation of electronic properties [41]. The vibrational frequencies were evaluated at the same theoretical level and the obtained positive frequencies confirmed the interaction between imprinting template and functional monomer. Furthermore, the binding energy of the monomer–template complex was determined by the following formula:

ΔE = E_complex_ − (E_template_ + E_monomer_). (1)

### 2.4. Preparation of GCE/MOF

The Cu BTC octahedral frameworks were directly prepared on the surface of GCE using copper sulfate salts in a three-phase manner. Prior to this process, the GCE was completely polished with a 0.05 µm alumina slurry and then ultrasonic in a 50% ethanol solution for 5 min to clean the surface impurities. Cu nanoparticles were then electrodeposited on the GCE using a 15 mL solution (pH = 7.4) containing (NH4)_2_SO_4_ (0.5 mol L^−1^) and CuSO_4_ (0.01 mol L^−1^), applying an optimal stabilizing potential of −0.40 V (relative to Ag/AgCl) over 650 s. After this, a desirable Cu layer with an appropriate thickness was prepared on the GCE surface and GCE/Cu was obtained. To convert copper metal into copper hydroxide nanotubes, GCE/Cu was immersed into a newly prepared solution containing 2.5 mL NaOH (10 mol L^−1^), 1.5 mL (NH_4_)_2_S_2_O_8_ (1 mol L^−1^), and 6.3 mL ultrapure water, without the addition of any other solvents. After 180 s of reaction, the color of the electrode surface turned dark blue, indicating the formation of the GCE/Cu(OH)_2_ nanotubes.

Then, the GCE/Cu(OH)_2_ nanotubes were immersed into a newly prepared linker solution containing 0.2 g H_3_BTC in 9.5 mL ethanol and 3.5 mL ultrapure water for 330 s to obtain Cu-BTC MOF. Finally, the modified electrode was rinsed with deionized water to eliminate the residual MOF microstructures and dried under nitrogen gas. The obtained GCE/MOF had a distinctly greenish tint. 

### 2.5. Preparation of GCE/MOF/MIP

To form an electro-polymerized cortisol MIP layer on the surface of the pre prepared GCE/MOF, a 10 mL Tris buffer solution (0.01 mol L^−1^ Tris buffer, 0.1 mol L^−1^ KCl, pH = 7.4) containing monomer o-PD, template cortisol, cross-linker EGDMA, and Prussian blue nanoparticles was obtained under optimized conditions. The automated laboratory glass battery was completely wrapped in aluminum foil to prevent self-polymerization, and the prepared GCE/MOF was inserted into the battery. Immediately, 17 cycles of solution electro polymerization were performed within the potential range of −0.6 to 0.6 V at a scanning rate of 21 mV s^−1^ (The condition was optimized by experimental design). Ultimately, the electrodes were immersed in a solution containing 3 mol L^−1^ KOH and pure ethyl alcohol (*V*/*V* = 5:1), and stirred for 13 min until the template molecules were completely eluted. The non-imprinted polymer (NIP)-modified electrode was prepared in practically the same way without adding cortisol. 

### 2.6. Experimental Design

The Chemometric Agile Tool (CAT) was used to design the mathematical model [42,43]. In multivariate analysis, the optimum conditions for the GCE/MOF/MIP electrode preparation were evaluated by varying different factors involved in the preparation process and the performance of the sensor. The experimental design was applied for screening the most important variables. In detail, the experimental plan consisted of conducting the experiments, estimating the importance of coefficients, and predicting responses and then validating the model. More specifically, a full factor design (2k design, k = 4) was applied to study the effect of four different factors on sensor performance. The preparation factors were the concentration of the templates and functional monomers. The selected conditions were the number of voltammetry cycles during the polymerization process and the time needed to elute the template molecules. These factors were studied at two levels (Table 1).

The concentration levels of the templates and functional monomers were fixed at specific ratios, as they were effective in generating the imprinting cavities. Typically, the development of imprinting materials requires a ratio of 1:2 of functional monomers to templates. Furthermore, the number of cycles applied for electro-polymerization was also considered to investigate whether the thickness of the MIP film would affect the sensor sensitivity. The elution time should be less than 15 min to avoid damaging the MIP film. The polynomial equation of 24 factors, where the coefficients represent the weights of linear and interaction terms between each considered factor, is shown below.

Y = b_0_ + b_1_X_1_ + b_2_X_2_ + b_3_X_3_ + b_4_X_4_ + b_12_X_1_X_2_ + b_13_X_1_X_3_ + b_14_X_1_X_4_ + b_23_X_2_X_3_ + b_24_X_2_X_4_ + b_34_X_3_X_4_ + b_123_X_1_X_2_X_3_ + b_124_X_1_X_2_X_4_ + b_134_X_1_X_3_X_4_ + b_234_X_2_X_3_X_4_ + b_1234_X_1_X_2_X_3_X_4_ (2)

In Equation (2), b_0_, b_i_ (i: 1→4), and b_ij_ (j: 1→4) represent the correlation coefficients of the constant term, linear term, and interaction term, respectively. Therefore, the experimental plan was randomly carried out, with a total of 19 experiments.

### 2.7. Fabrication of the Wearable Sensor

**Figure 1:**
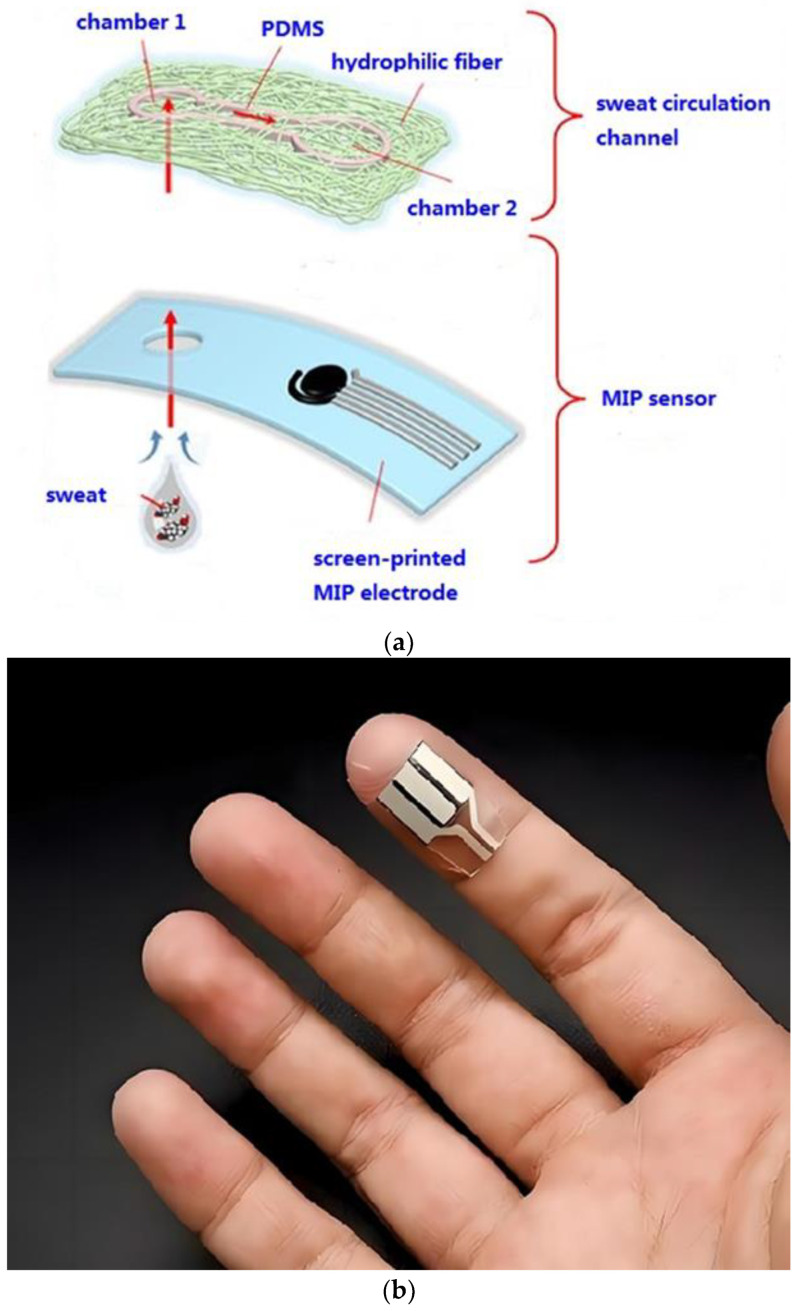
(**a**) Schematic illustration of the microfluid chip; (**b**) full view of the wearable sensor.

The wearable sensor consisted of the manufactured GCE/MOF/MIP electrode for electrochemical sensing, an Ag/AgCl reference electrode, an auxiliary electrode, and a microfluidic chip for sweat extraction (Figure 1a). When sweat was secreted, it passed through the inlet chamber 1 and was absorbed by the upper microfluidic chip due to its high porosity and hydrophilicity. Then, the sweat was automatically transmitted to chamber 2. The sweat in chamber 2 then served as an electrolyte, where the cortisol molecules were specifically recognized by the MIP and occupied some of the imprinting cavities. The decrease in the imprinting cavities could block the electron transfer of the in-built probes, resulting in changes in the response current. Ultimately, this sensor was integrated with a flexible circuit board which can be comfortably fitted on a human body (Figure 1b). The circuit board acted as a transducer and the results were transferred to a cell phone via Bluetooth.

### 2.8. Electrochemical Detection and Performance Test

During the electrochemical detection, a pair of redox peaks originated from the built-in Prussian blue probe was observed in blank PBS, but they showed an obvious decrease after the probe had been incubated with cortisol due to the occupation of imprinting cavities caused by the rebinding between cortisol and GCE/MOF/MIP. This showed that the rebinding of cortisol hindered the electron transfer, thereby suppressing the redox current. The response current decreased with the increase in cortisol concentration and a linear relationship between the current decrease and the cortisol concentration was achieved.

The human body research was performed according to the human body research protocol approved by the Medical Ethics Committee of Southwest University of Science and Technology. During indoor exercise (stationary exercise bike) at 7 a.m. and 7 p.m., the volunteers were evaluated by wearing sensors on their wrists. The in situ sweat measurement was taken after cycling for 15 min and incubating for 2 min, and the electrochemical data were then wirelessly transported to a Smartphone. To verify the accuracy of the wearable sensor, the results were compared with traditional human ELISA by ex situ tests of freshly collected sweat samples.

## 3. Results and Discussion

### 3.1. Computational Studies of the Interaction between Monomer and Cortisol

**Figure 2:**
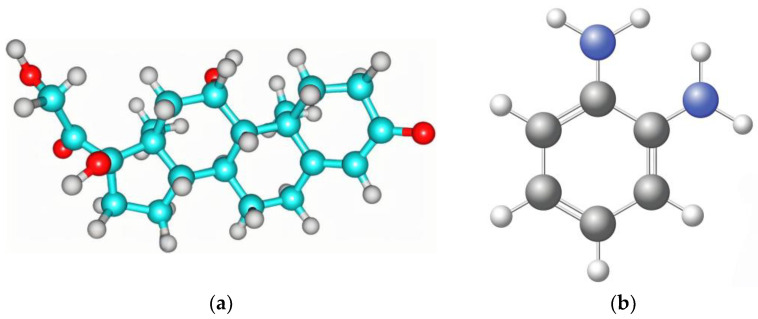

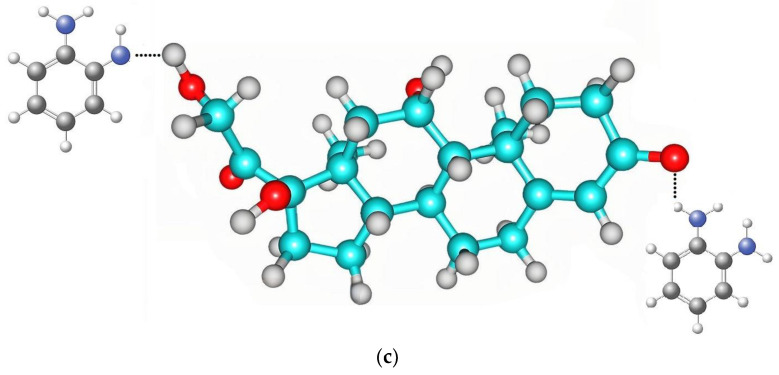
Optimized structure of (**a**) cortisol; (**b**) o-PD and (**c**) template–monomer complex.

Because of its biocompatibility, mechanical stability, and ability to form a thin and compact polymer matrix to provide a quick and stable sensing response, o-PD was chosen as the functional monomer for the molecular imprinting. The interaction between o-PD and the template cortisol was investigated by computational studies. Density functional theory at the ωB97XD/6–31 G (d,p) was used to calculate ΔE. The binding energies of the complex (Table 2) illustrate that the favorable stability of the o-PD-cortisol complex might be related to the strong hydrogen bonds formed between the nitrogen of o-PD and the hydrogen and oxygen of cortisol (Figure 2).

### 3.2. Interpretation of the Experimental Design 

The electrochemical synthesis of MIP was studied to investigate the influence of various factors on the performance of the sensor in terms of sensitivity. Therefore, a full factor design was carried out to identify the main factors affecting the performance of the GCE/MOF/MIP sensor. The response affected by those factors was the sensitivity of the sensor when the template was evaluated in the range of 1.0 and 50 nmol L^−1^. The following formula was used to calculate the weights of the coefficients evaluated by the model.

S = 0.559 + 0.197X_1_ + 0.039X_2_ + 0.073X_3_ + 0.302X_4_ + 0.058X_1_X_2_ − 0.013X_1_X_3_ + 0.133X_1_X_4_ + 0.055X_2_X_3_ − 0.107X_2_X_4_ − 0.156X_3_X_4_ + 0.113X_1_X_2_X_3_X_4_. (3)

**Figure 3:**
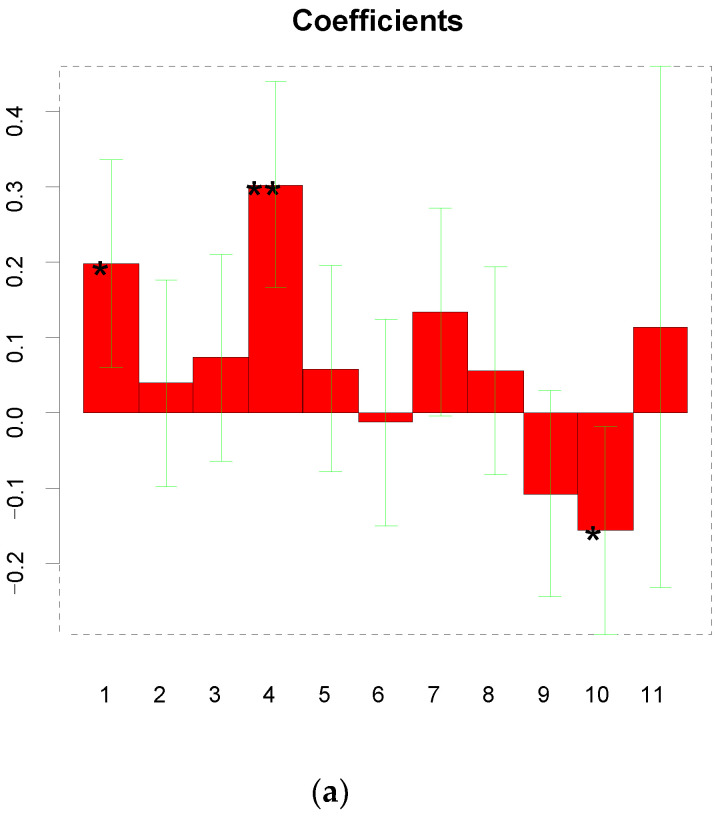

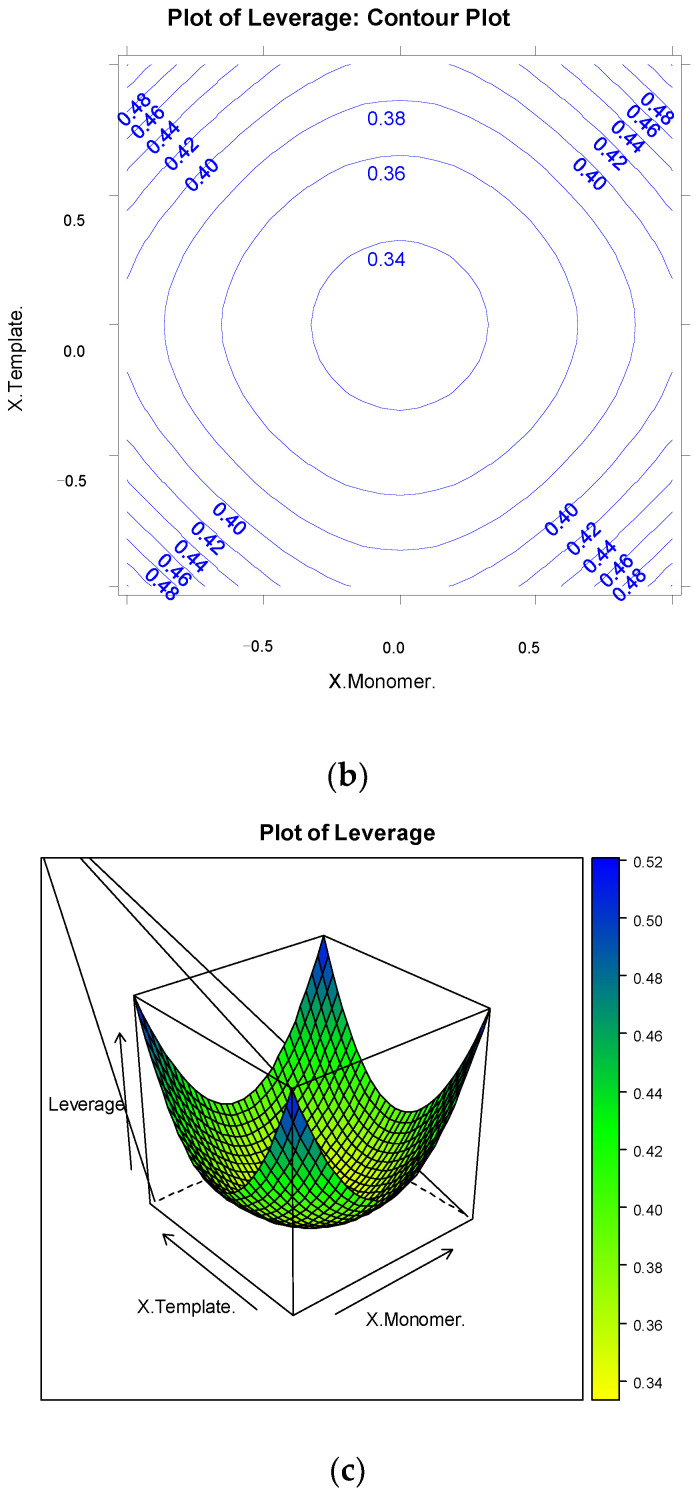
(**a**) Significance of the obtained coefficients; (**b**) contour plot of the leverage; and (**c**) 3D plot of the leverage surface. Note: “*” represents that the coefficient is significant at the level of 0.05 and “**” represents that the coefficient is significant at the level of 0.01.

In Equation (3), X_1_, X_2_, X_3_, and X_4_ were the concentration of monomers, the concentration of template molecules, the number of cycles applied for the electro-polymerization, and the time for elution, respectively. S is the sensitivity of the sensor, representing the value of responses. The results show that the more important factors are the concentration of the monomer (X_1_) and the elution time (X_2_). The coefficients and their significance are presented in Figure 3a. Furthermore, the goodness of the experimental matrix was evaluated by studying the proposed dispersion matrix, which showed a value of 0 for all the off-diagonal elements and a small value on the diagonal. This means that the variance of the coefficients is small and thus all the coefficients can be estimated with a good precision. The trajectory of the matrix reveals a more compact term about the goodness of the data. In addition, the inflation factors show the quality of each term in the model. To be specific, values less than four are considered acceptable. Finally, our experimental matrix reveals a good quality.

After evaluating the results, there seemed to be a strong correlation between the monomer concentration and the template concentration (Figure 3b,c). During the polymerization process, the appropriate template concentration ratio should be selected to guarantee the correct orientation of the functional monomers around the templates. In fact, the sensitivity was higher when the ratio of the monomer concentration to the template concentration was 1:2. At the last stage of preparing the GCE/MOF/MIP, the template molecules were eluted by exposing the modified electrode to a solution containing 3 mol L^−1^ KOH and pure ethyl alcohol. In line with the results, 13 min of elution time was used to further evaluate the sensor performance. The factors that affected the growth of the electro-synthetic membrane was not obvious, which allowed us to select the number of cycles based on the optimal stability of MOF/MIP on the electrode surface.

**Figure 4:**
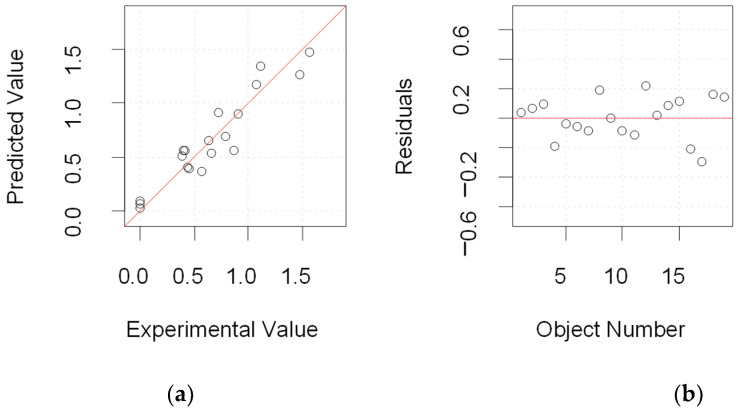
(**a**) Prediction proposed using the experimental design and (**b**) fitting with the experimental results.

In summary, in order to achieve the maximum sensitivity, the optimal experimental conditions were (1) monomer concentration: 3 mmol L^−1^; (2) template concentration: 6 mmol L^−1^, (3) CV cycles: 17; and (4) elution time: 13 min. Under these optimized conditions, additional experiments were carried out which fit into the prediction model. Figure 4 shows the prediction of the model and the fitting with the experimental values. The results showed that the experimental data were almost entirely consistent with the predicted values, verifying the effectiveness of the mathematical model.

### 3.3. Characterization of the Proposed GCE/MOF/MIP

#### 3.3.1. Optical Profilometry

**Figure 5:**
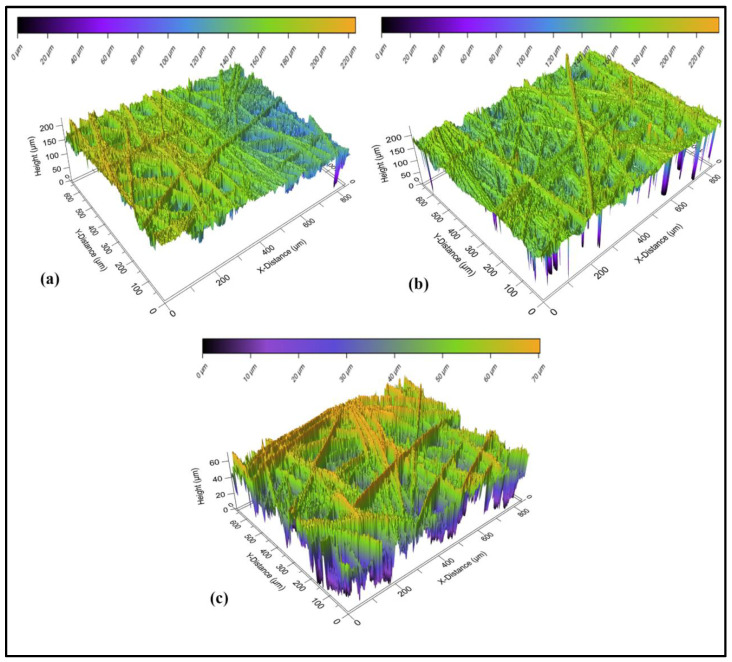
Three-dimensional images from optical profilometer: (**a**) GCE/MOF/NIP, (**b**) GCE/MOF/MIP before elution, and (**c**) GCE/MOF/MIP after elution.

Optical profilometry is a technique for the rapid and non-destructive analysis of surface morphology without touching the surface [44,45]. The study of surface roughness could verify the effectiveness of a modification and the information related to the changes in geometric surface area. The surfaces of GCE/MOF/NIP and GCE/MOF/MIP before and after elution were analyzed by an optical profilometer (Figure 5). The Root Mean Square value, a representation of the surface roughness, was found to be 10.31 μm for the GCE/MOF/NIP, 10.37 μm for the GCE/MOF/MIP before elution and 11.76 μm for the GCE/MOF/MIP after elution, respectively. It can be seen that, after eluting the template molecules, the specific surface area significantly increased. The high negative skewness value of the GCE/MOF/MIP after elution confirms the formation of cavities after the template removal. The surface area (S_a_) and skewness (S_sk_) values at the micro-scale level are displayed in Table 3.

#### 3.3.2. Electrochemical Characterization

**Figure 6:**
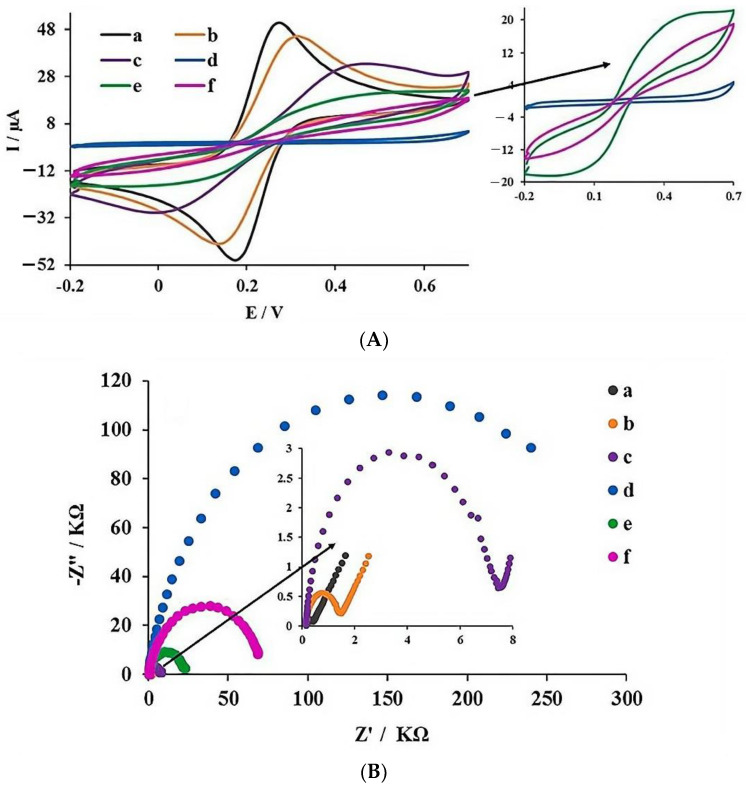
(**A**) CV curves (insert: the magnification of the last three steps) and (**B**) Nyquist curves (insert: the magnification of the first three steps). (a) GCE, (b) GCE/MOF, (c) GCE/MOF/pre-polymerization, (d) GCE/MOF/MIP before elution, (e) GCE/MOF/MIP after elution and (f) GCE/MOF/MIP after incubation of cortisol solution.

Since most MIPs are non-conductive materials, the electron transfer on the electrode surface is significantly affected and the detection sensitivity is notably reduced [46,47]. Therefore, the use of a conductive MOF can not only increase the electrode surface area, but also form a conductive layer, which is important in developing a hybrid imprinting sensor. Cyclic voltammetry (CV) and electrochemical impedance spectroscopy (EIS) were used to study electrochemical behavior when 5 mmol L^−1^ [Fe(CN)_6_]^3−^/^4−^ (in 0.1 mmol L^−1^ KCl solution) was used as the probe. In impedance measurement, the semicircle diameter of the high-frequency region is related to the charge transfer resistance (R_ct_) while the linear part of the low-frequency region depicts the diffusion process [48]. From the voltammograms shown in Figure 6, the existence of a pair of clear redox peaks confirms the reversible electrochemical process occurring on the bare GCE (a in Figure 6A). A decrease in the peak current is observed after the immobilization of MOF on the GCE surface (b in Figure 6A). After the immersion of the MOF-modified electrode in the pre-polymerization solution, a decrease in the peak current was observed, which could be attributed to the spatial potential barrier generated by the pre-polymerization solution (c in Figure 6A). Subsequently, a sharp decrease in the peak current was observed due to the increase in the spatial barrier after electro-polymerization (d in Figure 6A). After the elution of the template molecules, an increase in peak current appears (e in Figure 6A), which might be due to the improvement of electron transfer after the generation of the imprinting sites. Ultimately, after the incubation of GCE/MOF/MIP with a cortisol solution, a decrease in the perk current was observed due to the selective extraction of the cortisol molecules by the MIP cavities and the increase in the spatial restriction on the electrode surface. This whole process proves the successful preparation of the GCE/MOF/MIP.

To further characterize the sensing surface, Nyquist curves at different stages of the preparation process were recorded by EIS (Figure 6B). A comparison of the charge transfer resistance (R_ct_) values of GCE (a in Figure 6B) and MOF/GCE (b in Figure 6B) shows that the R_ct_ value of GCE/MOF (1.49 kΩ) is higher than that of bare GCE (0.4 kΩ). This could be ascribed to the reduced electron transfer between the redox probe and the GCE/MOF surface. In addition, the R_ct_ value further increased to 7.55 kΩ after the immersion of GCE/MOF in the pre-polymerization complex (c in Figure 6B), which illustrates that the electron transfer on the electrode surface is more difficult and steric hindrance is greater. R_ct_ value increases obviously after electro-polymerization (d in Figure 6B), which could be ascribed to there being more steric hindrance on the electrode surface. After elution, a significantly decrease in R_ct_ was observed (e in Figure 6B), due to the reduction in the spatial restriction after the removal of the template from the MIP complex. In the last stage, the R_ct_ value increased to 68.78 kΩ after the incubation of the cortisol solution, which might be because that the cortisol molecules are selectively caught by the imprinting sites and electron transfer is hindered more. These EIS results are in perfect agreement with the observations in the CV characterization, demonstrating the successful preparation of the sensor interface.

#### 3.3.3. Characterization of Microfluidic Sample Acquisition

In order to realize the spontaneous collection and routing of sweat, a nanofiber-based microfluidic chip with strong hydrophilicity was adopted. The microfluidic chip was fabricated by writing on the nanofiber membrane with PDMS ink. Due to the high porosity of the nanofiber membrane, PDMS ink can flow into the interior of the membrane to form channels and chambers. The fluid velocity was regulated by adjusting the width of the microfluidic channel. The microfluidic chip with an opening and a sensing region aligned with the inlet and the reaction chamber was directly fabricated on the GCE/MOF/MIP electrode and integrated into a wearable device. When sweat is secreted, due to the outstanding hydrophilicity of the nanofiber membrane, it can be instantly absorbed by the microfluidic chip through the inlet and quickly transferred to the sensing region along the micro-channel.

**Figure 7:**
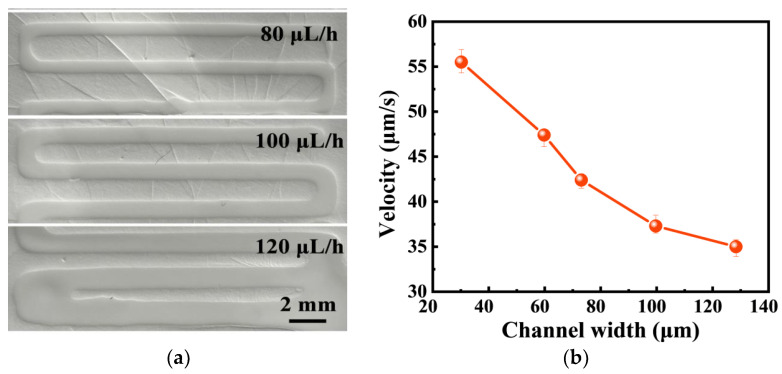
(**a**) Photographs of the microfluidic channels with different ink flow rates. (**b**) Fluid velocity of microfluidic channels with varying widths.

The successful implementation of the wearable sensor was an important requirement for obtaining a sufficient number of samples to assess the analyte concentration, while maintaining direct and continuous sampling to reduce the environmental factors that primarily lead to pollution and uncontrolled transportation. Bodily fluids must be collected and transferred to the wearable biosensor surface with minimal delay to generate timely and accurate sensor responses. In this work, microfluidic technology was used to realize rapid sampling. After the optimization of the ink flow rates (Figure 7a) and channel width, to obtain the shortest response time (Figure 7b), a 30 μm wide channel was created by laser patterning on a 70 μm thick single-sided medical grade tape, and connected to the GCE/MOF/MIP sensor through a double-sided medical grade tape, which enabled passive capillary driven fluid control and could be used as a sample bank to hold a volume sweat over 100 μL. This amount is sufficient to provide a stable sensor reading. The results showed that, by using microfluidic, a tiny volume of sweat sample can be collected on the sensor surface to produce the same quality of response as bulk solution analysis.

### 3.4. Real-Time Detection of Sweat Cortisol by Prepared Wearable GCE/MOF/MIP Sensor

**Figure 8:**
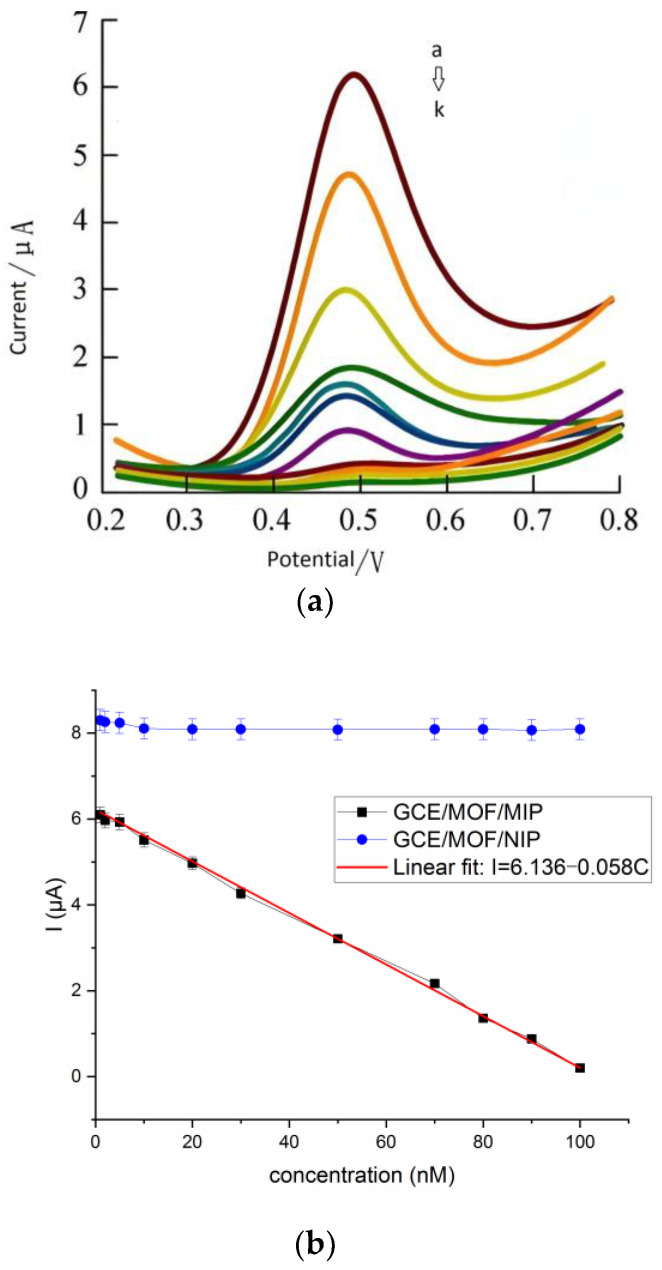
(**a**) The DPV responses of the wearable sensor to different concentrations of cortisol. Cortisol concentration: a: 1.0 nM; b: 10 nM; c: 100 nM; d: 200 nM; e: 300 nM; f: 400 nM; g: 500 nM; h: 600 nM; i: 700 nM; j: 800 nM; k: 1000 nM. (**b**) the relationship between cortisol concentrations and I (blue: GCE/MOF/NIP sensor; black: GCE/MOF/MIP sensor; red: linear fit).

As illustrated in Figure 8a, the response current decreases with the increase in the cortisol concentration, indicating that the extract of the template cortisol can hinder electron transfer. A linear relationship between the peak current and the cortisol concentration is established in the range of 0.01 to 1000 nmol L^−1^ with a linear regression equation of I (μA) = 6.136 – 0.058 C (nmol L^−1^) (R^2^ = 0.993) (Figure 8b). The detection limit was calculated to be 0.0027 nmol L^−1^ (S/N = 3), which is lower than those reported in most studies [49,50,51]. This excellent sensitivity comes from the high specific surface area of the MOF substrate. Considering that the physiological level of cortisol in human sweat is between 0.02 and 0.5 μmol L^−1^, this wearable sensor can meet the requirements of actual cortisol detection. Furthermore, the current change was much lower when the GCE/MOF/NIP sensor was used to detect the same cortisol solution. This illustrates that the results obtained by the GCE/MOF/MIP sensor were attributable to the specific binding of MIP. 

To evaluate the feasibility of the sensor, the standard addition method was used to detect the cortisol concentration in artificial sweat. As shown in Table 4, the recoveries are between 94.7 and 103.6%, and the RSD is under 5%, indicating the high reliability of the proposed GCE/MOF/MIP sensor.

### 3.5. Selectivity and Durability of the Prosed GCE/MOF/MIP Sensor

**Figure 9:**
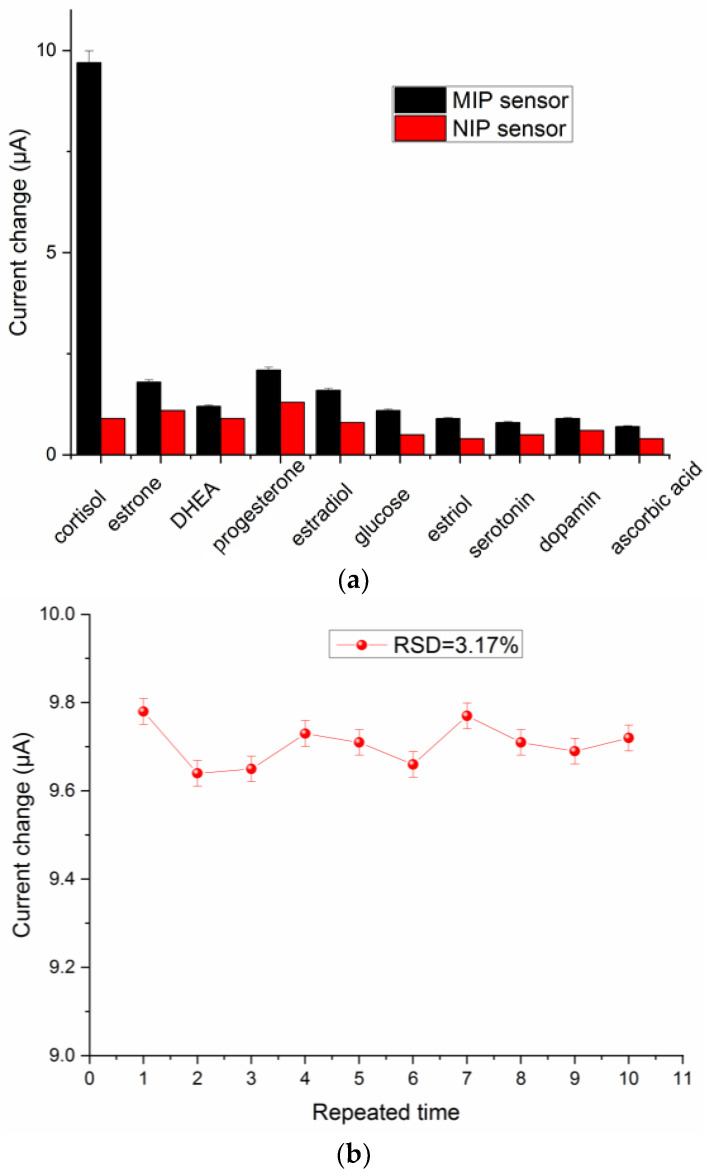
(**a**) Selectivity of the GCE/MOF/MIP and GCE/MOF/NIP sensors; (**b**) repeatability of the GCE/MOF/MIP sensor. All experiments were performed using three parallel tests.

To assess the specificity of the proposed GCE/MOF/MIP sensor, the effects of common steroid hormones and interfering substrate were studied, including estrone, DHEA, progesterone, estradiol, glucose, estriol, serotonin, dopamine, and ascorbic acid. As illustrated in Figure 9a, the sensor displays a significant cortisol output signal, but the observed responses in the presence of interference are negligible. In contrast, the GCE/MOF/NIP sensor did not show obvious difference between cortisol and the interference analogies. This excellent resistance to interferences comes from the imprinting sites which are perfectly matched with the target molecules, allowing the GCE/MOF/MIP sensor to detect the cortisol concentration in complex matrices accurately.

In addition, the repeatability of the sensor was checked through a continuous measurement of cortisol, with a relative standard deviation (RSD) of 3.17% (Figure 9b). To study the reproducibility, five different sensors prepared using the same procedure were used for the same cortisol detection, yielding a RSD of 4.36%. Furthermore, for long-term stability tests, the sensor was kept in a refrigerator and a 50 nmol L^−1^ cortisol solution was measured every three days. The results showed that the decrease in the response current was negligible, and 94.3% of the initial signal was maintained after 30 days. To sum up, the developed sensor has the advantages of high sensitivity, excellent specificity, and good stability and reproducibility, showing broad application potential.

The analytical performance of the proposed sensor was compared with some other reported sensors (Table 5). The comparison shows that this work exhibits superior performance in several aspects, such as a broad dynamic range, a low detection limit and high selectivity.

## 4. Conclusions

In this work, an electrochemical wearable sensor that could be integrated with an MOF/MIP for the on-body monitoring of cortisol in sweat was prepared. MOF is an ideal substrate for MIP polymerization due to its high porosity and interconnected 3D structure. Moreover, it exhibited an excellent electrochemical sensor performance because it can accelerate electron transfer. The proposed sensor performs well in terms of selectivity, accuracy, and stability, which enables it an excellent measurement of cortisol over a wide detection range. This sensor is hopeful to provide a versatile strategy for designing wearable sensors for the real-time monitoring of sweat biomarkers other than cortisol.

## Figures and Tables

**Table 1:** Factors and related levels considered in the experimental design.

Factors	Units	Low	High
Monomer concentration (X_1_)	mmol L^−1^	0.05	3
Cortisol concentration (X_2_)	mmol L^−1^	0.1	6
Number of CV cycles (X_3_)		7	39
Elution time (X_4_)	min	3	13

**Table 2:** Binding Energy of o-PD-cortisol Complex.

Molecule	Energy (a.u.)	Energy (eV)	ΔE (a.u)	ΔE (eV)
O-phenylenediamine	−796.34267 ± 0.63241	−20,356.87821 ± 16.37264		
Cortisol	−2193.66542 ± 1.38562	−61,573.38429 ± 23.58513		
Complex	−2915.22996 ± 1.94675	−79,166.75309 ± 26.79324	74.77813 ± 0.073281	2013.50941 ± 1.079312

**Table 3:** Microporous roughness parameters of MOF/NIP electrode, MOF/MIP electrode before elution, and MOF/MIP electrode after elution.

Electrode	S_a (_µm)	S_sk_ (µm)
GCE/MOF/NIP	10.31 ± 0.24	−2.871 ± 0.031
GCE/MOF/MIP before elution	10.37 ± 0.24	−4.116 ± 0.039
GCE/MOF/MIP after elution	11.76 ± 0.26	−5.378 ± 0.045

**Table 4:** Determination of cortisol in artificial sweat by standard addition method (S/N = 3).

Sample	Spiked (nM)	Found (nM)	Recovery (%)	RSD (%)
1	1	0.947	94.7	4.15
2	10	10.12	101.2	2.86
3	100	103.6	103.6	4.03
4	500	493.7	98.7	4.17
5	1000	1013.4	101.3	3.95

**Table 5:** Comparison of this proposed with other reported cortisol sensors.

Electrode	Sensing Unit	Sample	Detection Range (nM)	LOD (nM)	Ref
ITO	Ferrocene tagged antibodies	Saliva	0.028–137.9	0.028	[52]
Reduced graphene oxide	Anti-cortisol antibodies	Saliva/sweat	0.2758–551.7	0.2758	[53]
Carbon	Carbon nanotubes/ metalloporphyrins	Saliva	0.00005–100	0.00005	[54]
Graphene + AuNPs-triamcinolone	Aptamer	Serum	0.0827–27,500	0.275	[55]
GCE/MOF/MIP	MIP	Sweat	0.0027–1000	0.0027	This work

## Data Availability

Data are unavailable due to privacy.
